# Obituary

**Published:** 1878-02

**Authors:** 


### OBITUARY.

Dr. Geo. J. Barclay, of Pittsburg, Pa., died suddenly of internal hemorrhage in New York, at the residence of Dr. W.

H. Atkinson, at 12.15 a. m., January 22d, '78, in the fifty-third year of his age. Dr. Barclay, a courteous gentleman, and one of our best operators, was of those who can ill be spared by either the profession or the public.

